# Melatonin Rescues Triclosan-Disrupted Porcine Oocyte Meiosis via Suppression of p53-Mediated Apoptosis

**DOI:** 10.3390/ani15213193

**Published:** 2025-11-03

**Authors:** Jiaxin Duan, Ning Zhao, Shibin Wang, Xinyu Li, Bugao Li, Guoqing Cao

**Affiliations:** 1 College of Animal Science, Shanxi Agricultural University, Jinzhong 030801, China; djxsxnydx@sxau.edu.cn (J.D.); z18434764303n@163.com (N.Z.); wang0907bin@163.com (S.W.); lxy02806@163.com (X.L.); 2 Shanxi Key Laboratory of Animal Genetics Resource Utilization and Breeding, Jinzhong 030801, China

**Keywords:** triclosan, melatonin, porcine oocytes, apoptosis, p53

## Abstract

**Simple Summary:**

Triclosan (TCS), a widely used antimicrobial agent present in numerous consumer products such as soaps and toothpaste, has raised growing concerns regarding its potential adverse effects on fertility. In this study, we employed porcine oocytes as a model to investigate how TCS affects their development and whether melatonin, a natural hormone, can offer protection. We found that TCS exposure severely disrupts oocyte meiotic maturation and furthermore triggers apoptosis. Importantly, we discovered that melatonin effectively reverses this damage by suppressing the p53 pathway, an intrinsic stress-response system that would otherwise signal the oocytes to arrest their development and undergo programmed cell death. Our findings elucidate a mechanism through which melatonin protects oocyte quality and suggest its potential as a protective agent against the detrimental effects of common environmental chemicals on female fertility.

**Abstract:**

Triclosan (TCS), a widely used environmental antimicrobial agent, poses potential risks to female reproductive health, yet its toxic effects on oocyte maturation remain inadequately characterized. In this study, we established an in vitro maturation (IVM) model of porcine oocytes to investigate TCS-induced meiotic impairment and to evaluate the rescuing effects of melatonin (MT), an endogenous indoleamine with potent antioxidant and anti-apoptotic activities. Our results demonstrated that TCS exposure significantly disrupted oocyte maturation, as evidenced by suppressed polar body extrusion and compromised cumulus expansion. Furthermore, TCS triggered early apoptosis. Proteomic analysis revealed that the p53 signaling pathway was significantly dysregulated by TCS exposure. Notably, co-treatment with MT during IVM effectively restored meiotic progression, attenuated apoptosis, and rebalanced the disrupted proteomic profile. Mechanistic investigation, validated by Western blotting, confirmed that TCS upregulated p53 and downregulated its downstream cell cycle effector CCNB1 while concurrently altering the ratio of apoptosis-related proteins BAX/BCL-2. Melatonin treatment effectively normalized the expression of these key proteins (p53, CCNB1, BAX, and BCL-2). These findings illustrate that MT rescues TCS-impaired oocyte quality through p53-dependent suppression of apoptosis and restoration of meiotic progression, providing new insights into potential strategies for mitigating environmental pollutant-induced reproductive damage.

## 1. Introduction

The pervasive presence of synthetic chemicals in the environment poses a significant threat to global public health. The reproductive system is particularly vulnerable to this threat. A major concern stems from endocrine-disrupting chemicals (EDCs), which can impair female fertility by interfering with hormonal homeostasis and cellular redox balance [1]. For instance, epidemiological evidence has demonstrated an association between exposure to endocrine-disrupting chemicals, including bisphenol A and phthalates, and an increased risk of polycystic ovary syndrome (PCOS) [2,3]. Furthermore, recent studies have further indicated that diisobutyl phthalate (DiBP) impairs oocyte quality by disrupting meiotic progression, mitochondrial function, and inducing oxidative stress and apoptosis [4]. Given that oocyte quality is a decisive factor for female fertility and early embryonic development, it is crucial to understand the mechanistic impact of EDCs. Therefore, investigating the effects of specific EDCs, particularly the widely used antimicrobial triclosan, on oocyte quality is essential for developing strategies to protect reproductive health. For this investigation, porcine oocytes were selected as a physiologically relevant model due to their established similarities to human oocytes and documented sensitivity to environmental toxicants [5,6].

Triclosan (TCS), a man-made broad-spectrum biocide, is commonly utilized in a range of consumer goods, including hygiene items, medical apparatus, and domestic cleaning agents [7]. However, its environmental persistence, bioaccumulation potential, and endocrine-disrupting effects have raised significant health concerns [8,9]. Accumulating evidence indicates that TCS promotes oxidative stress, compromises mitochondrial integrity, and induces apoptotic cell death across diverse cell types, particularly within reproductive cells [10,11]. Specifically, it has been shown to impair oocyte maturation—a prerequisite for successful fertilization [12]. Given the documented reproductive toxicity and widespread human exposure to TCS, elucidating its mechanisms and exploring protective strategies in physiologically relevant models like the porcine oocyte are paramount.

To counteract these detrimental effects, melatonin (MT), an endogenous indoleamine with potent antioxidant, anti-inflammatory, and anti-apoptotic activities, has emerged as a promising therapeutic agent [13]. In the context of reproductive biology, MT contributes to improved oocyte quality through its ability to eliminate reactive oxygen species (ROS), preserve mitochondrial structural and functional integrity, and reduce stress on the endoplasmic reticulum (ER) [14]. Its efficacy in alleviating damage from environmental toxins like bisphenols and pesticides is well-documented [15,16,17,18]. Based on this established protective profile, we hypothesized that MT could also protect oocytes against TCS-induced defects. However, the specific mechanisms by which MT might mitigate TCS-induced oocyte damage, particularly in physiologically relevant porcine models, remain poorly understood.

The p53 signaling pathway serves as a central regulator in cellular physiology, orchestrating DNA damage response, apoptosis, and cell cycle arrest [19]. For instance, studies in turbot oocytes have demonstrated that melatonin inhibits apoptosis and enhances antioxidant capacity through the p53 and caspase-dependent pathways, thereby improving meiotic maturation [20]. Similarly, research in porcine endometrial stromal cells showed that the flavonoid rutin alleviates mycotoxin-induced ferroptosis and mitochondrial damage via p53 signaling, underscoring its protective role under toxic stress [21]. Given that oxidative stress and organelle dysfunction are known activators of p53 [22,23], and considering its central role in deciding cellular fate under stress, we hypothesized that the p53 pathway might be a critical integrator of TCS-induced toxicity in oocytes. Although oxidative stress, organelle dysfunction, and apoptosis have been implicated in TCS-induced oocyte impairment [12], the upstream signaling pathways that integrate these cytotoxic effects, particularly the role of the p53 pathway, are not defined. Furthermore, whether the protective effect of melatonin against such insults operates through the modulation of this specific pathway remains to be elucidated.

Based on the established reproductive toxicity of TCS and the documented protective capacity of MT, this study sought to investigate the impact of TCS on porcine oocyte maturation and to evaluate the potential rescuing effects of MT. We specifically hypothesized that TCS impairs oocyte quality primarily by inducing apoptosis and that MT confers protection through the modulation of key cellular signaling pathways. To test this, a combination of fluorescence staining, Western blotting, and proteomic analysis was employed to assess nuclear maturation and apoptotic pathways in oocytes exposed to TCS with or without MT co-treatment. Our findings elucidate a central mechanism of TCS-induced oocyte defects and MT-mediated protection, offering insights into potential strategies for ameliorating reproductive impairments caused by environmental pollutants.

## 2. Materials and Methods

### 2.1. Ethics Statement

Ovarian samples from sows were obtained from animals processed at commercial slaughter facilities in Shanxi Province. Therefore, formal ethical review was not applicable to this work.

### 2.2. Antibodies and Chemicals

Chemical reagents were obtained from GlpBio (Montclair, CA, USA). Hormonal preparations were supplied by Ningbo No. 2 Hormone Factory (Ningbo, China), and staining kits were purchased from Beyotime Biotechnology (Shanghai, China). Primary antibodies targeting p53, BAX, BCL-2, and CCNB1 were sourced from Proteintech (Wuhan, China), while the anti-GAPDH antibody was provided by Servicebio (Wuhan, China).

### 2.3. In Vitro Culture of Porcine Oocytes

The ovaries were transported to the laboratory within 1.5 h in phosphate-buffered saline (PBS) pre-warmed to 38.5 °C and supplemented with 1% penicillin-streptomycin [24]. Healthy follicles were aspirated using a 11-gauge needle to collect follicular fluid [25]. After centrifugation, the follicular fluid was allowed to settle by gravity in a 10 mL tube for at least 20 min. The supernatant was then carefully removed, and oocytes with intact cumulus-oocyte complexes (COCs) and uniform cytoplasm were selected under a stereomicroscope (Nikon, Tokyo, Japan) for further use.

Groups of 10 COCs were cultured in 100 µL of maturation medium prepared with M199 (Gibco, Waltham, MA, USA) and enriched with 0.91 mmol/L sodium pyruvate, 0.57 mmol/L L-cysteine, 3.05 mmol/L glucose, 1 mg/mL polyvinyl alcohol, 10 ng/mL epidermal growth factor, 10% FBS (Gibco, Waltham, MA, USA), 10% porcine follicular fluid, and gonadotropins (10 IU/mL PMSG and hCG, plus 10 IU/mL FSH). TCS was prepared as a stock solution in Dimethyl Sulfoxide (DMSO) and subsequently diluted in the culture medium to the desired working concentrations. The control group (0 μmol/L TCS) received an equal volume of DMSO. MT was prepared as a 100 mmol/L stock solution in DMSO and further diluted in maturation medium to obtain working concentrations of 0.002, 0.2, and 20 μmol/L. The DMSO content was kept constant at 0.1% (*v*/*v*) in all experimental groups. For combination treatments, MT was introduced together with TCS. Cultures were maintained at 38.5 °C under 5% CO_2_ for 44 h [26].

### 2.4. Assessment of Maturation in Oocytes

Following 44 h of in vitro culture, the extent of cumulus expansion in COCs was examined under a stereomicroscope (Nikon, Tokyo, Japan). The degree of cumulus expansion was classified into three distinct categories using a subjective scoring system, which was based on established methods and defined by the increase in diameter relative to denuded oocytes [27,28].

For nuclear maturation assessment, cumulus cells were then removed by gentle pipetting in PBS containing 1 mg/mL hyaluronidase. After washing, denuded oocytes were stained with Hoechst 33342 for chromatin observation. After washing, denuded oocytes were stained with Hoechst 33342 for chromatin observation. Observation under a fluorescence microscope (Nikon, Tokyo, Japan) revealed that metaphase II (MII) stage oocytes, considered mature, were identified by the presence of two distinct bright chromatin spots. The first polar body extrusion rate was calculated as follows: PBE rate = (Number of oocytes with the first polar body/Total number of oocytes examined) × 100%.

### 2.5. Analysis of Early Apoptosis

Early apoptosis in porcine oocytes was assessed using an Annexin V-FITC apoptosis detection kit. Briefly, after 44 h of in vitro maturation under respective treatment conditions, oocytes were washed three times in PBS and subsequently incubated in a working solution containing Annexin V-FITC (diluted 1:15 in binding buffer) for 40 min at room temperature in the dark. Following incubation, the oocytes were washed again three times with fresh binding buffer to remove unbound dye. Fluorescent signals were immediately captured using a fluorescence microscope (Nikon, Tokyo, Japan). Oocytes exhibiting a clear green fluorescent signal on the membrane were considered to be in the early stages of apoptosis. The apoptosis rate was calculated as the percentage of Annexin V-positive oocytes relative to the total number of oocytes examined in each group. Each experiment was independently repeated at least three times.

### 2.6. Bioinformatics and Proteomic Analysis

DIA (Data-Independent Acquisition) proteomic sequencing was performed on porcine oocytes from the Control, TCS-exposed, and TCS + MT treatment groups. Functional profiling and pathway enrichment analysis of the differentially expressed proteins (DEPs) were performed using the Gene Ontology (GO) and Kyoto Encyclopedia of Genes and Genomes (KEGG) databases. Visualization of the proteomic results—including heatmaps, volcano plots, and functional enrichment graphs—was performed using R language (v4.1.2) and the OmicShare online platform. The mass spectrometry proteomics data have been deposited to the ProteomeXchange Consortium via the PRIDE partner repository with the dataset identifier PXD068303 [29].

### 2.7. Western Blotting

A total of sixty oocytes were lysed in 10 μL of RIPA Lysis Buffer supplemented with 1% PMSF. A volume equivalent to one-fifth of the resultant lysate of SDS-PAGE loading buffer was added, followed by boiling the mixture for 10 min. The extracted proteins were resolved by SDS-PAGE and transferred onto nitrocellulose (NC) membranes for subsequent assays. Membranes were blocked for 15 min, then incubated overnight at 4 °C with the designated primary antibodies. After primary incubation, membranes were exposed for 1 h at room temperature to HRP-conjugated goat anti-rabbit secondary antibodies (LI-COR Bioscience, Lincoln, NE, USA) with gentle agitation. Following washing steps, protein bands were detected on X-ray film using the ECL Plus system (LI-COR Biosciences, Lincoln, NE, USA).

### 2.8. Statistical Analysis

Each experiment was conducted independently on at least three separate occasions. Statistical evaluation was carried out using GraphPad Prism software (version 9.5). The results are expressed as the mean ± standard error of the mean (SEM). For comparisons involving more than two groups, one-way analysis of variance (ANOVA) was applied, followed by Tukey’s post hoc test for pairwise group comparisons. The *p*-value less than 0.05 was considered to be significant.

## 3. Results

### 3.1. Melatonin Rescues TCS-Impaired Nuclear Maturation in Porcine Oocytes

**Figure 1:**
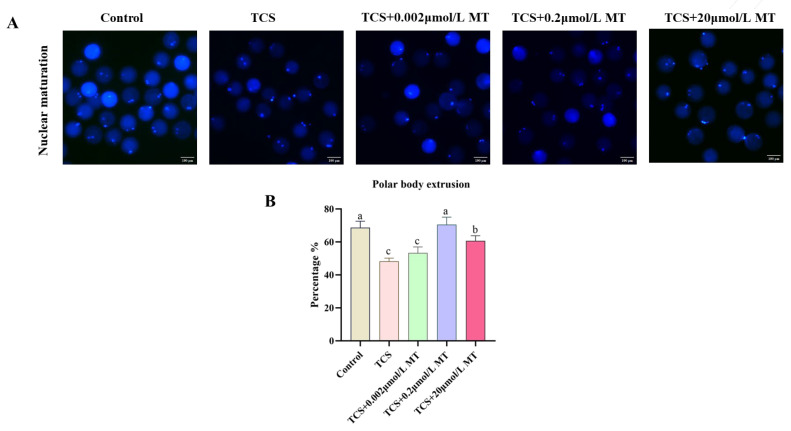
Melatonin restores TCS-impaired meiotic progression in porcine oocytes. (**A**) Representative fluorescence images of the first polar body in the control, TCS, and TCS + MT groups (0.002, 0.2, and 20 μmol/L). Scale bar = 100 μm. (**B**) Expulsion rate of the first polar body in the control, TCS, and TCS + MT groups (0.002, 0.2, and 20 μmol/L). Different letters above bars indicate significant differences (*p* < 0.05).

In order to confirm the detrimental effects of TCS [12] and the protective role of melatonin on porcine oocyte maturation, the present study further investigated the protective role of MT. As shown in Figure 1A,B, TCS exposure significantly suppressed polar body extrusion (PBE), while co-treatment with MT effectively reversed this impairment. Specifically, compared to the TCS-only group (48.24% ± 1.12%), the PBE rates were significantly restored to 53.27% ± 2.12% in the 0.002 μmol/L MT group, 70.52% ± 2.60% and 60.71% ± 1.77% in the 0.2 μmol/L and 20 μmol/L MT co-treatment groups, respectively (all *p* < 0.05). Given that 0.2 μmol/L MT exhibited the most prominent rescuing effect, this concentration was selected for all subsequent rescue experiments.

### 3.2. Melatonin Restores TCS-Suppressed Cumulus Expansion in Porcine Oocytes

**Figure 2:**
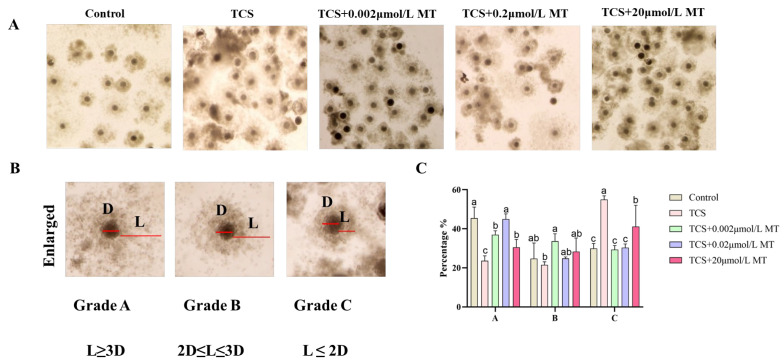
Melatonin counteracts TCS-induced suppression of cumulus expansion in porcine oocytes. (**A**) Representative morphology of COC expansion in the control, TCS, and TCS + MT groups (0.002, 0.2, and 20 μmol/L). (**B**) Classification of COC expansion: Grade A (fully expanded), Grade B (partially expanded), and Grade C (poorly expanded). (**C**) Statistical analysis of COC expansion grades in oocytes from the control, TCS, and TCS + MT groups (0.002, 0.2, and 20 μmol/L). Different letters above bars indicate significant differences (*p* < 0.05).

Consistent with its adverse effect on nuclear maturation, TCS exposure also significantly suppressed cumulus expansion, a key morphological indicator of oocyte developmental competence. As illustrated in Figure 2A, co-treatment with 0.2 μmol/L MT, the most effective concentration identified in the maturation assay, substantially alleviated this defect. The proportion of Grade A (fully expanded) COCs was significantly higher in the MT co-treatment group (42.92% ± 1.56%) compared to the TCS-only group (23.62% ± 1.46%) (*p* < 0.05). Correspondingly, the percentage of Grade C (poorly expanded) COCs decreased significantly from 54.92% ± 1.12% in the TCS group to 30.31% ± 1.11% in the TCS + MT group (*p* < 0.05; Figure 2C). These results collectively demonstrate that 0.2 μmol/L MT effectively restores TCS-impaired cumulus expansion in porcine oocytes.

### 3.3. Melatonin Suppresses Apoptosis in TCS-Exposed Porcine Oocytes

**Figure 3:**
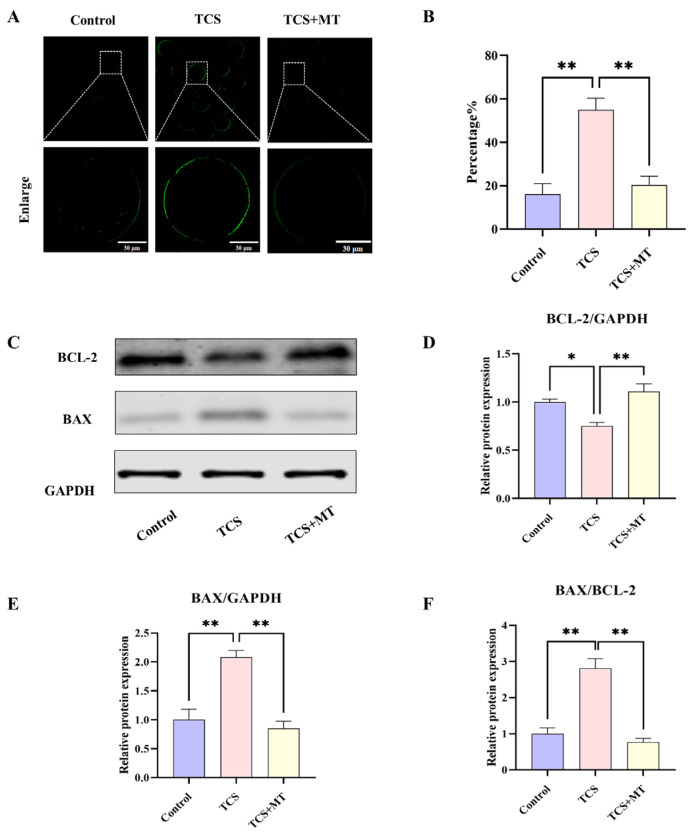
Melatonin suppresses TCS-induced apoptosis in porcine oocytes. (**A**) Annexin V-FITC staining for early apoptosis in Control, TCS, and TCS + MT oocytes. Scale bar = 100 μm. (**B**) Apoptosis rate quantification across groups. (**C**) Representative Western blot images showing the protein levels of BCL-2, BAX, and the loading control GAPDH. (**D**–**F**) Quantification of relative BAX and BCL-2 protein levels (normalized to GAPDH), and the resulting BAX/BCL-2 ratio. * *p* < 0.05, ** *p* < 0.01.

To investigate the apoptotic mechanisms induced by TCS, early apoptosis was evaluated using Annexin V-FITC staining. Control oocytes exhibited minimal fluorescence, with an apoptosis rate of 16.11% ± 4.87%, whereas TCS exposure resulted in pronounced phosphatidylserine externalization and a significant increase in the apoptosis rate to 55.02% ± 5.31% (*p* < 0.01; Figure 3A,B). This TCS-induced apoptosis was effectively attenuated by melatonin co-treatment, which restored the apoptosis rate to 20.45% ± 3.98% (*p* < 0.01), a level comparable to the control.

Western blot analysis indicated that TCS altered the expression profile of BCL-2 family proteins, reducing the anti-apoptotic protein BCL-2 (*p* < 0.05) and elevating the pro-apoptotic protein BAX (*p* < 0.01), which led to a marked increase in the BAX/BCL-2 ratio (*p* < 0.01). Administration of MT significantly increased BCL-2 levels (*p* < 0.01), decreased BAX expression (*p* < 0.01), and restored the BAX/BCL-2 ratio (*p* < 0.01), indicating a strong suppressive effect on the mitochondrial pathway of apoptosis (Figure 3C–F).

### 3.4. Proteomic Analysis Implicates p53 Signaling in MT’s Protection

**Figure 4:**
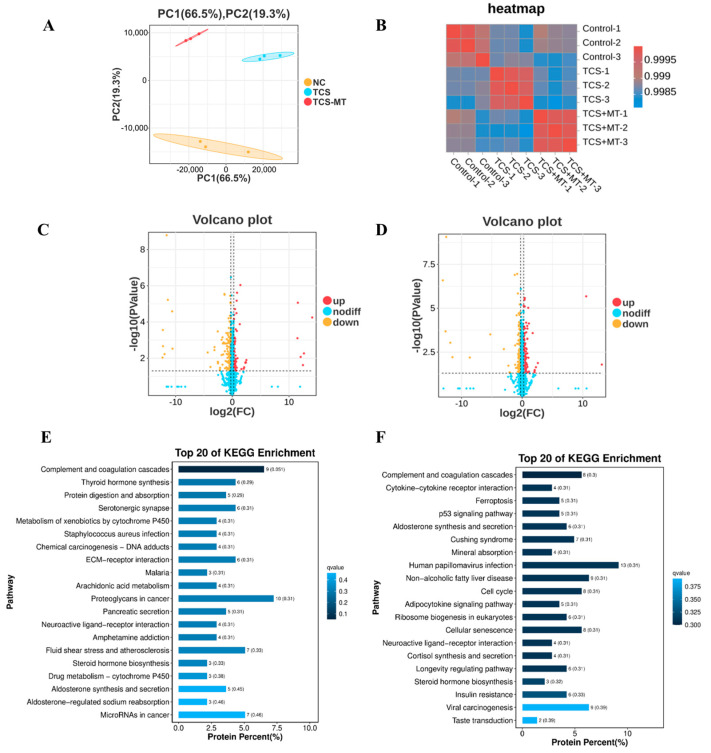
Melatonin attenuates TCS-induced toxicity through regulation of the p53 pathway. (**A**) Principal component analysis (PCA) of the proteomic profiles across all experimental groups. (**B**) Heatmap depicting Pearson correlation coefficients between individual samples. (**C**) Volcano plot displaying differentially expressed proteins (DEPs) in the Control vs. TCS comparison. (**D**) Volcano plot of DEPs in the TCS vs. TCS + MT comparison. (**E**) KEGG pathway enrichment analysis of DEPs identified from the Control vs. TCS comparison. (**F**) KEGG pathway enrichment analysis of DEPs identified from the TCS vs. TCS + MT comparison.

To investigate the impact of TCS and the rescue effect of MT at the proteomic level, we conducted quantitative proteomic profiling. PCA results showed a clear segregation of the three experimental groups, and the high degree of consistency among biological replicates within each group indicated excellent reproducibility of the data (Figure 4A). Comparative analysis identified 289 differentially expressed proteins (DEPs) after TCS exposure (95 upregulated, 194 downregulated). MT treatment significantly modulated the expression of 342 DEPs compared to the TCS group (164 upregulated, 178 downregulated) (Figure 4C,D).

KEGG pathway enrichment analysis revealed that these DEPs were primarily involved in pathways related to cancer, metabolism, and signal transduction. After significance filtering, the p53 signaling pathway was identified as one of the most significantly enriched pathways, suggesting its critical role in the biological processes under investigation (Figure 4E,F).

### 3.5. Melatonin Mitigates TCS Toxicity by Normalizing p53 Pathway Activation

**Figure 5:**
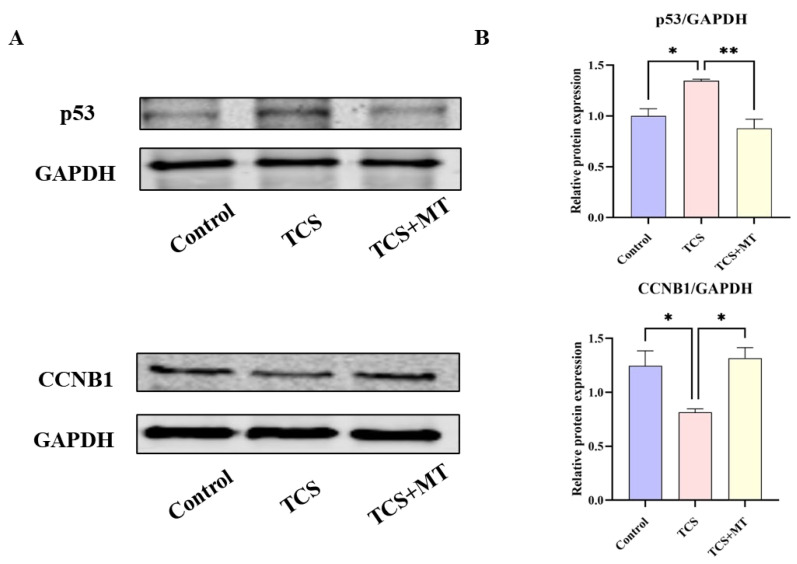
Melatonin restores the TCS-induced dysregulation of p53 and CCNB1 expression. (**A**) Representative immunoblot images showing the protein expression levels of p53, CCNB1, and the loading control GAPDH in oocytes from the indicated groups. (**B**) Densitometric quantification of relative p53 and CCNB1 protein levels normalized to GAPDH. * *p* < 0.05, ** *p* < 0.01.

To validate the role of the p53 signaling pathway in TCS-induced toxicity and MT-mediated protection, we quantified the protein expression levels of p53 and CCNB1 using Western blot analysis normalized to GAPDH (Figure 5A). Compared with the control group, TCS exposure significantly upregulated p53 expression (*p* < 0.05) and downregulated CCNB1 expression (*p* < 0.05). Importantly, MT treatment restored both p53 (*p* < 0.01) and CCNB1 (*p* < 0.05) expression to physiological levels, suggesting that reactivation of the p53 pathway by melatonin alleviates TCS-induced cellular damage (Figure 5B). This biochemical validation supports our earlier KEGG pathway enrichment analysis and confirms that p53 signaling is a central mechanism underlying the protective effects of melatonin.

### 3.6. p53 Inhibition Attenuates TCS-Induced Apoptosis but Abrogates Melatonin’s Protective Effect on Apoptotic Signaling

**Figure 6:**
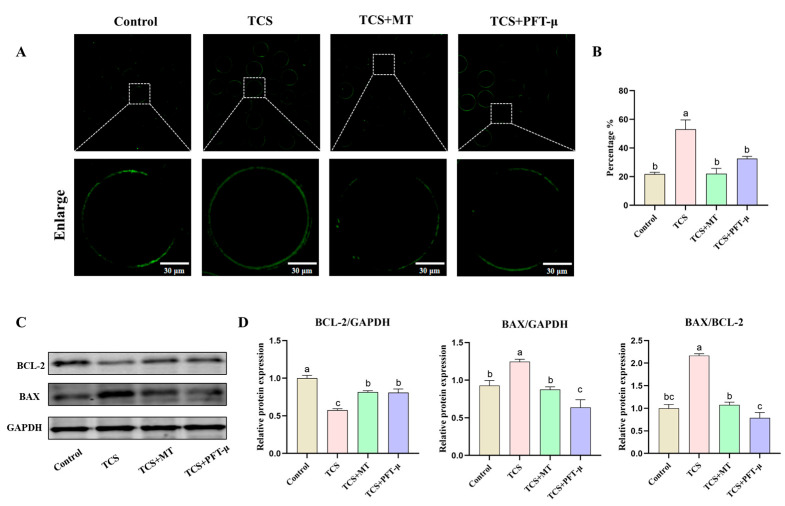
Pharmacological inhibition of p53 attenuates TCS-induced apoptosis and disrupts melatonin-mediated restoration of apoptotic protein balance. (**A**) Representative fluorescence images of early apoptosis (Annexin V-FITC staining) in oocytes from the Control, TCS, TCS + MT, and TCS + PFT-μ groups. Scale bar = 30 μm. (**B**) Rate of early apoptosis across different treatment groups. (**C**) Representative Western blot images of BCL-2, BAX, and GAPDH protein expression. (**D**) Densitometric analysis of relative BAX protein levels, BCL-2 protein levels, and the BAX/BCL-2 ratio, normalized to GAPDH. Different letters above bars indicate significant differences (*p* < 0.05).

We next evaluated the contribution of the p53 pathway to TCS-induced apoptosis. To specifically inhibit p53 activity, we employed PFT-μ, a pharmacological inhibitor that blocks p53-mediated transcriptional activation and apoptosis. Assessment by Annexin V-FITC staining revealed that TCS exposure significantly increased the early apoptosis rate to 53.18% ± 6.47% compared to 21.74% ± 1.28% in the control group (Figure 6A,B). This TCS-induced apoptotic response was significantly mitigated by pharmacological inhibition of p53 with PFT-μ, which reduced the apoptosis rate to 32.67% ± 1.51% (*p* < 0.05). Notably, co-treatment with melatonin (TCS + MT group) demonstrated a potent anti-apoptotic effect, restoring the apoptosis rate to a level comparable with the control (22.12% ± 3.64%). Western blot analysis of the apoptosis-related proteins BCL-2 and BAX (with GAPDH as the internal control; Figure 6C) revealed that pharmacological inhibition of p53 signaling with PFT-μ significantly elevated the BAX/BCL-2 ratio (*p* < 0.05; Figure 6D). This rebound effect indicates a reinstatement of pro-apoptotic signaling and confirms that the anti-apoptotic effect of MT is dependent on functional p53 pathway activity. Together, these phenotypic and molecular findings underscore the critical role of the p53 pathway in mediating TCS-induced oocyte damage and establish that MT mitigates this toxicity primarily through p53-dependent mechanisms.

### 3.7. Pharmacological Inhibition of p53 Partially Rescues TCS-Impaired Nuclear Maturation

**Figure 7:**
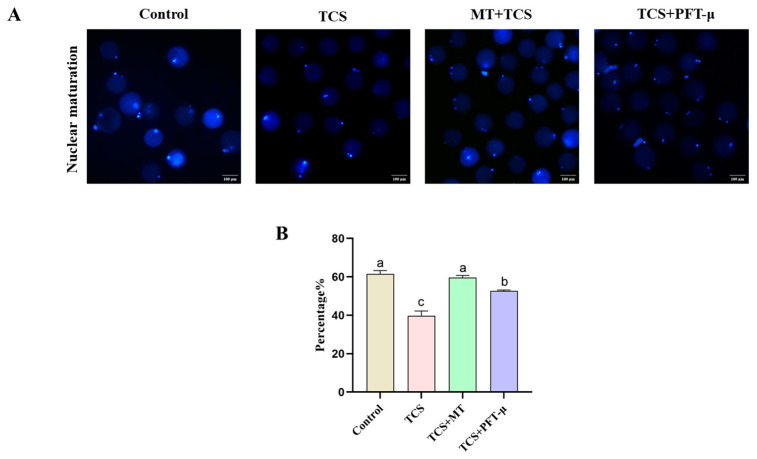
p53 inhibition partially rescues TCS-impaired nuclear maturation in porcine oocytes. (**A**) Representative fluorescence images of the first polar body in oocytes from the Control, TCS, TCS + MT, and TCS + PFT-μ groups. Scale bar = 100 μm. (**B**) First polar body extrusion rate in oocytes from the indicated groups. Different letters above bars indicate significant differences (*p* < 0.05).

To determine whether the p53 pathway mediates the detrimental effects of TCS on meiotic progression, we assessed the rate of first polar body extrusion following treatment with the p53-specific inhibitor PFT-μ. TCS exposure significantly suppressed the polar body extrusion rate to 39.78% ± 2.48% compared to 61.56% ± 1.74% in the control group (*p* < 0.05). Co-treatment with melatonin (TCS + MT group) effectively restored maturation, with the rate recovering to 59.65% ± 1.14%. Notably, the addition of PFT-μ (TCS + PFT-μ group) also led to a partial but significant rescue of the extrusion rate (52.70% ± 0.48%) compared to the TCS-only group (*p* < 0.05; Figure 7A,B). These findings indicate that p53 activation is a key contributor to the meiotic arrest caused by TCS exposure, and its inhibition can partially alleviate the maturation defect.

## 4. Discussion

TCS, a synthetic broad-spectrum antimicrobial agent prevalent in personal care and medical products, has become a widespread environmental contaminant, raising significant concerns regarding its potential impacts on female reproductive health [30,31,32]. While its toxicity in various somatic cells is increasingly recognized, the specific mechanisms by which TCS impairs the function of the female germline—particularly oocyte maturation—remain inadequately defined. Similarly, although MT has demonstrated broad efficacy in protecting against reproductive toxins [15,16,17], its potential to counteract TCS-induced oocyte damage and the underlying mechanisms are largely unexplored. To this end, we employed porcine oocytes as a physiologically relevant model, justified by their established similarities to human oocytes [6] and documented sensitivity to environmental toxicants [28,33,34]. Our results systematically demonstrate that TCS exposure severely disrupts meiotic maturation by activating the mitochondrial pathway of apoptosis. Crucially, we identify that melatonin rescues oocyte quality primarily by restoring the homeostasis of the p53 signaling pathway, thereby revealing a novel and specific mechanistic axis for MT-mediated protection against environmental reproductive toxicants.

The maturation of oocytes is commonly assessed through two critical morphological indicators: the expansion of the surrounding cumulus cells and the extrusion of the first polar body, both of which are tightly correlated with cytoplasmic maturation and developmental competence [35,36]. In the present study, TCS exposure significantly compromised oocyte maturation, as demonstrated by markedly reduced polar body extrusion rates and severely impaired cumulus expansion, consistent with previous reports of TCS-induced oocyte defects. Furthermore, TCS triggered apoptosis through the dysregulation of Bcl-2 family proteins, elevating Bax and suppressing Bcl-2. Notably, co-treatment with MT effectively restored these morphological and molecular deficits. This protective role of MT aligns with its well-established efficacy in countering pollutant-induced damage through multifaceted mechanisms—including potent antioxidant defense, anti-apoptotic regulation, and mitochondrial function maintenance [15,16,37]. Thus, our findings confirm that MT can effectively counteract TCS-induced impairments in oocyte maturation and apoptosis, providing a foundation for investigating the specific upstream signaling pathways orchestrating this protection.

Building upon the observed morphological deficits, we next investigated whether apoptosis contributed to TCS-induced meiotic failure. Our analysis revealed a significant increase in early apoptosis following TCS exposure, coupled with a pronounced dysregulation of BCL-2 family proteins. Specifically, TCS treatment markedly elevated the expression of the pro-apoptotic protein BAX while suppressing the anti-apoptotic protein BCL-2, resulting in a substantially increased BAX/BCL-2 ratio. These findings align with established apoptotic pathways and are consistent with reports of TCS-triggered apoptotic cell death in other reproductive models [12,38,39]. Notably, co-treatment with melatonin effectively attenuated early apoptosis and restored the balance between BAX and BCL-2. The anti-apoptotic potency of MT observed in our porcine oocyte model aligns with its documented efficacy in protecting germ cells against diverse environmental stressors. Mechanistically, melatonin is known to exert its anti-apoptotic effects through multiple pathways, including scavenging reactive oxygen species (ROS), maintaining mitochondrial membrane potential, and regulating key apoptotic mediators such as the BCL-2/BAX ratio and caspase cascades [16,40,41,42]. These consistent findings across different models underscore MT’s pleiotropic protective nature. However, while these established mechanisms provide an important context, the specific upstream signaling pathways through which MT coordinates this integrated protective response in TCS-exposed oocytes remained to be elucidated. This gap in understanding prompted our subsequent proteomic investigation to identify the key regulators involved.

The potent anti-apoptotic activity of MT observed in the present study is consistent with its well-established role in mitigating cellular stress. While its capacity to reduce ROS and alleviate oxidative stress in oocytes is well-documented [43,44,45], our findings directly demonstrate that MT suppresses TCS-induced apoptosis in porcine oocytes, as evidenced by the restored BAX/BCL-2 ratio and diminished Annexin V signal. While these established mechanisms provide a plausible framework for MT’s action, the specific upstream signaling pathways responsible for orchestrating this protection in our model remained unclear. This gap in understanding prompted a proteomic investigation to identify the key regulators involved.

To elucidate the overarching mechanisms through which melatonin counteracts TCS-induced toxicity, we employed an unbiased quantitative proteomic approach, which identified the p53 signaling pathway as a central hub. The activation of this pathway provides a unified explanation for the two major phenotypic outcomes observed: meiotic arrest and apoptosis. As a classic tumor suppressor, p53 plays a well-established role in regulating cell cycle progression, DNA repair, and apoptosis [46,47]. Our findings position p53 as a critical sensor of TCS-induced cellular stress in oocytes, consistent with its recognized function in integrating diverse stress signals, including oxidative stress and DNA damage, in other cellular contexts [48,49]. The concomitant dysregulation of its downstream effector CCNB1 provides a direct mechanistic link to the observed meiotic arrest. Being a crucial regulator of the G2/M transition in both mitotic and meiotic cell cycles, CCNB1 depletion directly impairs cell cycle progression [50,51], Consequently, the TCS-p53-CCNB1 axis identified here offers a coherent molecular explanation for the maturation failure: TCS activates the p53 pathway, which in turn suppresses CCNB1, thereby arresting meiotic progression. Melatonin’s intervention, by normalizing this regulatory axis, effectively releases this cell cycle brake.

To establish causality beyond correlation, we conducted functional studies using the specific p53 inhibitor PFT-μ. The significant attenuation of TCS-induced defects in nuclear maturation and apoptosis upon p53 inhibition provides direct evidence for p53’s central role in mediating TCS toxicity. More importantly, the complete abolition of MT’s restorative benefits by PFT-μ unequivocally demonstrates that a functional p53 pathway is indispensable for melatonin-mediated protection. This dependency relationship reveals that MT’s action is not merely permissive but requires active p53 signaling to execute its rescue effects. Collectively, our integrated multi-omics and functional data establish that the p53 signaling pathway serves as a causal and central mediator orchestrating both TCS-induced oocyte damage and MT-conferred protection. The melatonin-mediated recovery of p53 and CCNB1 homeostasis represents a prominent molecular mechanism that effectively bridges the gap between initial cellular insult and final phenotypic outcome. However, while our study clarifies the downstream events, the upstream triggers of p53 activation upon TCS exposure—and specifically whether it is activated through the DNA damage response or through DNA damage-independent pathways such as those mediated by stress-activated kinases [52]—remain to be fully elucidated. Determining the precise route of p53 activation represents an important focus for future research aimed at completing the mechanistic picture of TCS reproductive toxicity.

## 5. Conclusions

In conclusion, our study establishes that TCS compromises porcine oocyte quality by activating the p53 signaling pathway, which leads to meiotic arrest through CCNB1 downregulation and triggers mitochondria-dependent apoptosis. Conversely, melatonin effectively rescues these defects by normalizing p53 pathway activity, thereby restoring meiotic progression and suppressing apoptosis. Crucially, we demonstrate that MT’s protective efficacy is contingent upon functional p53 signaling. These findings not only delineate a novel p53-dependent mechanism for MT-mediated protection against environmental reprotoxicants but also highlight the potential of targeting this pathway to safeguard female fertility.

## Data Availability

The data presented in this study are available on request from the corresponding author.
